# The Functionality of the Cysteinyl Leukotriene Receptor 1 (CysLTR1) in the Lung by Metabolomics Analysis of Bronchoalveolar Lavage Fluid

**DOI:** 10.21203/rs.3.rs-8052995/v1

**Published:** 2025-11-24

**Authors:** Wilson Bamise Adeosun, Sibongiseni KL. Poswayo, Suraj P. Parihar, Du Toit Loots

**Affiliations:** Biomedical and Molecular Metabolism (BioMMet), North‒West University, Potchefstroom, South Africa; Division of Medical Microbiology, Institute of Infectious Diseases and Molecular Medicine (IDM), Faculty of Health Sciences, University of Cape Town, Cape Town, South Africa; Division of Medical Microbiology, Institute of Infectious Diseases and Molecular Medicine (IDM), Faculty of Health Sciences, University of Cape Town, Cape Town, South Africa; Centre for Infectious Disease Research in Africa (CIDRI-Africa), Faculty of Health Sciences, University of Cape Town, Cape Town, South Africa; Biomedical and Molecular Metabolism (BioMMet), North‒West University, Potchefstroom, South Africa

**Keywords:** cysteinyl leukotriene receptor 1, bronchoalveolar lavage fluid, metabolomics

## Abstract

**Introduction:**

The cysteinyl leukotriene receptor 1 (CysLTR1) is known as a potent lipid mediator with a well-established role in inflammatory regulation and lung disease. While its involvement in immune cell recruitment has been previously reported, its broader impact on pulmonary metabolism remains poorly understood.

**Objectives:**

The study aims to investigate the metabolic consequences of a CysLTR1 deletion in mice to elucidate its role in pulmonary metabolic homeostasis.

**Methods:**

Bronchoalveolar lavage fluid (BALF) was collected from CysLTR1 knockout (KO) and wild-type (WT) mice and analysed using standardized untargeted gas chromatography–time-of-flight mass spectrometry (GC-TOFMS) metabolomics.

**Results:**

Metabolomics analyses of the BALF collected from the CysLTR1 KO mice presented significantly reduced levels of glucose, glucosamine, and glyceric acid, indicating the role of the CysLTR in lung glucose uptake and consequently lung glycolysis and gluconeogenesis. This is further supported by reductions in myo-inositol and D-chiro-inositol, also supporting previous findings that this occurs due to insulin resistance. Consequential disruption of various glucose-dependent pathways, including the pentose phosphate pathway (reduced gluconic acid, sedoheptulose and xylose) and purine metabolism (reduced 1-methylinosine) indicates a consequential altered nucleotide turnover, and the significantly reduced concentrations of butanoic acid, decan-2-ol, and 1-hexadecanol, indicate changes to fatty acid metabolism in the lung, as a compensatory response to the initial glucose deficiency induced by the CysLTR1 KO. Lastly, the changes to mandelic acid, glutaric acid, tricarballylic acid, and decan-2-ol, furthermore, indicate the role of CysLTR1 in the composition/metabolism of the microbiome.

**Conclusion:**

This study expands our knowledge on the role of CysLTR1 beyond its role in immune regulation, that may later serve towards a better understanding of CysLTR1 associated lung diseases and in the development of improved therapeutic strategies.

## Introduction

Leukotrienes (LTs) and cysteinyl leukotrienes (CysLTs; LTC4, LTD4 and LTE4) are lipid mediators derived from arachidonic acid via the 5-lipoxygenase pathway, and function as regulators of inflammation, vascular permeability, and smooth muscle contraction (Kanaoka & Boyce 2004). Leukotrienes, especially leukotriene B4 (LTB4), regulate how macrophages respond to antigens, and are associated with inflammation. This phenomenon is important in the progression of various diseases, including atherosclerosis (Sánchez-Galán et al., 2009), obesity and type 2 diabetes (Filgueiras et al., 2015; Mothe-Satney et al., 2012). Considering the latter, the binding of LTB4 to its receptor results in various inflammatory responses, leading to insulin resistance. Interestingly, elevated concentrations of LTB4 in type 2 diabetes patients have also been shown to be correlated with cardiovascular autonomic dysfunction (Neves et al., 2020).

The cysteinyl leukotrienes function via the G protein-coupled receptors - CysLTR1, CysLTR2, and CysLTR3, and are best described for their role in various allergic and asthmatic responses and inflammation. For example, the CysLTs regulate the production of proinflammatory cytokines and adipokines, including interleukin 6 (IL-6), monocyte chemotactic protein 1, tumor necrosis factor-α (TNF-α), nuclear factor kappa B (NF-κB), and macrophage inflammatory protein 1 (Coffey et al., 2015; Filgueiras et al., 2015). Furthermore, CysTLR1 mediates inflammation during infection, and it has been demonstrated that mice lacking CysLTR1 have reduced granuloma, hepatic fibrosis, and liver enzyme release, which in turn is associated with an increased anti-inflammatory cytokine production. Furthermore, treating mice with montelukast (a CysLTR1 antagonist) in combination with praziquantel, resulted in a reduced cellular infiltration into the liver and a reduced egg burden during chronic *Schistosoma mansoni* infection, and a combination therapy of CysLTR1 inhibition and praziquantel, serves as a prophylactic treatment approach (Mosala et al., 2024). Eosinophils, mast cells, and chemosensory epithelial airway tuft cells, are also reportedly activated by CysLTs, leading to the type-2 immune responses, mucus secretion and exacerbation of pulmonary illnesses (Liu et al., 2025; Salimi et al., 2017). More recently, CysLTR1 inhibition has been shown to modulate intracellular glucose levels in retinal endothelial cells, pericytes and ARPE-19 cells, indicating a possible role in cellular glucose metabolism (Koller et al., 2025).

Bronchoalveolar lavage fluid (BALF) is an important biofluid used for researching changes to the lung microenvironment. The procedure involves washing the lower respiratory tract (bronchoalveolar space) with saline solution, which is then recovered. The solute content is composed mostly of alveolar macrophages, lymphocytes, neutrophils, proteins and primary lung metabolites (Kalidhindi et al., 2021; Sabounchi-Schütt et al., 2003). A metabolomics analysis of BALF would subsequently provide a detailed snapshot of the metabolic changes in the the lung due to a perturbation. This study subsequently investigated the role of CysLTR1 on the lung by comparing the metabolic changes associated with a CysLTR1 KO mouse model with that of a WT control, using GC-TOFMS metabolomics analysis of BALF samples.

## Materials and methods

### Reagents

Deionized water from a Millipore Milli-Q purification system was used throughout the study. Optimagrade acetonitrile was obtained from Fisher Scientific (Pittsburgh, USA). Unless otherwise stated, all other reagents and organic solvents used in this investigation were sourced from Sigma‒Aldrich (St. Louis, MO, USA).

#### Samples

CysLTR1-deficient (Cysltr1−/−) mice were generated by breeding heterozygous (Cysltr1−/+) animals on a C57BL/6 background. Dr. Frank Austen from Harvard Medical School generously provided CysLTR1 heterozygous mice. The mice were housed in ventilated cages under specific-pathogen-free conditions at the research animal facility of the UCT Faculty of Health Science. Mice aged 8–12 weeks were used, with sex matching, unless otherwise specified. The mice were terminally anesthetized, after which a catheter was inserted into the trachea and secured in place. A 1 mL syringe containing sterile saline solution was then connected to the catheter and gently injected. The solution was gently aspirated while the thorax of each mouse was massaged to collect BALF. Approximately 500–800 μL of BALF per mouse was transferred into 2 mL cryotubes, immediately snap-frozen in liquid nitrogen, and stored at − 80°C until analysis. All the animal studies adhered to the rigorous guidelines outlined in the South African National Standard for the Care and Use of Animals for Scientific Purposes (SANS 10386:2008). The study protocol was approved by the Animal Research Ethics Committee (AREC 022/024) at the Faculty of Health Science, University of Cape Town.

To monitor analytical reproducibility and ensure data quality, pooled QC samples were prepared by combining equal volume aliquots from each experimental BALF sample included in the study prior to extraction. The pooled QC sample was further aliquoted and treated as a biological sample, undergoing the same sample extraction, and derivatisation steps. Extraction blanks, went through the sample preparation, extraction and derivatisation steps, but did not contain any biological sample.

#### Sample preparation and extraction

A 100 μL volume of each BALF sample, was centrifuged at 10, 000 rpm for 10 minutes at room temperature, after the addition of 50 μL of a 3-phenylbutyric acid (Sigma‒Aldrich) internal standard (50ppm) and 300 μL of acetonitrile. The supernatant was subsequently evaporated to dryness before derivatization. The dried metabolic extract was oximated using 50 μL of methoxyamine hydrochloride in pyridine (15 mg/mL) (Merck, Darmstadt, Germany) at 50°C for 1 h, and then silylated using 50 μL of N, O-bis (trimethylsilyl) trifluoroacetamide (BSTFA) with 1% trimethyl chlorosilane (TMCS) (Sigma‒Aldrich, St. Louis, MO, USA) at 60°C for 1 h. The derivatized sample extracts were transferred to 2 mL glass vials containing 250 μL glass inserts.

One microliter of each sample extract was injected (1:1 split ratio) into a Pegasus BT GC-TOFMS instrument (Leco Corporation, St. Joseph, MI, USA), equipped with an Agilent 7890A gas chromatograph (Agilent, Atlanta, GA, USA) coupled to a time‒of-flight mass spectrometer (TOFMS) (Leco Corporation, St. Joseph, MI, USA). Separation was achieved via an Rxi-5-MS column (29.690 m, 0.25 mm internal diameter and 0.25 μm film thickness) (Restch GmbH & Co. KG, Haan, Germany). The front inlet temperature was held at a constant 270°C, the transfer line temperature at a constant 250°C, and the ion source temperature at a constant 200°C for the entire run. The initial GC oven temperature was set at 70°C for 1 min, followed by an increase of 5°C/min to a final temperature of 320°C, at which it was held for 3 min. The detector acquisition delay for each run was 420 s and was offset with a filament bias of − 70 eV. Spectra were collected from between 50 to 950 m/z at an acquisition rate of 20 spectra per second. Mass spectral deconvolution, peak alignment and peak identification were performed via Leco Corporation’s ChromaTOF software (version 4.71). Mass spectral deconvolution, peak deconvolution, peak alignment and identification were performed at a signal-to-noise ratio of 30, with a minimum of three apex peaks. To eliminate the effects of retention time shifts and create a data matrix containing the relative abundances of all the compounds present in all the samples, peaks with identical mass spectra and retention times were aligned using ChromaTOF. Mass fragmentation patterns and their respective retention times were screened against the National Institute of Standards and Technology (NIST) mass spectra library, as well as in-house libraries compiled from previously injected standards. Peak annotation was performed using a similarity threshold of at least 70%. All metabolite markers identified as described in section 2.5 were manually checked again by the analyst by comparing their retention times and mass fragment patterns to those of the libraries to confirm their identities before biological interpretation.

The extracted BALF samples were randomly injected, ensuring that any residual analytical variation was equally distributed among the groups, thereby minimizing potential bias. The prepared QCs, extraction blanks, and a FAMEs standard, were injected 3 times at the beginning, middle and end of the sample batch.

#### Statistical analysis and metabolite identification

The relative concentrations of 201 compounds comprising the combination of quality control (QT), WT and KO samples were statistically analyzed. Standard metabolomics data clean-up procedures were followed (including data pretreatment, spectral deconvolution, missing data input, data normalization (including log transformation) and batch effect correction) (Gromski et al., 2014; Sullivan & Feinn, 2012), and the cleaned data were further processed using various statistical analyses, including t-test, log 2-fold change (log2FC), and effect size analyses, to determine which metabolites contributed most significantly to the observed changes between the sample groups. A principal component analysis (PCA) was also conducted to determine the compounds contributing most to the observed variability and their relationships with the groups. All data analyses were performed via Metaboanalyst 6.0 (Pang et al., 2024).

## Results

### Raw data summary

A total of 936 compounds were detected via benchtop GC‒TOFMS. The dataset was meticulously cleaned to eliminate compounds without matching mass spectral entries in the libraries, and other artifact compounds of no biological relevance, resulting in the aforementioned 201 compounds (excluding the internal standard), which were subjected to the aforementioned statistical analyses.

**Figure 1:**
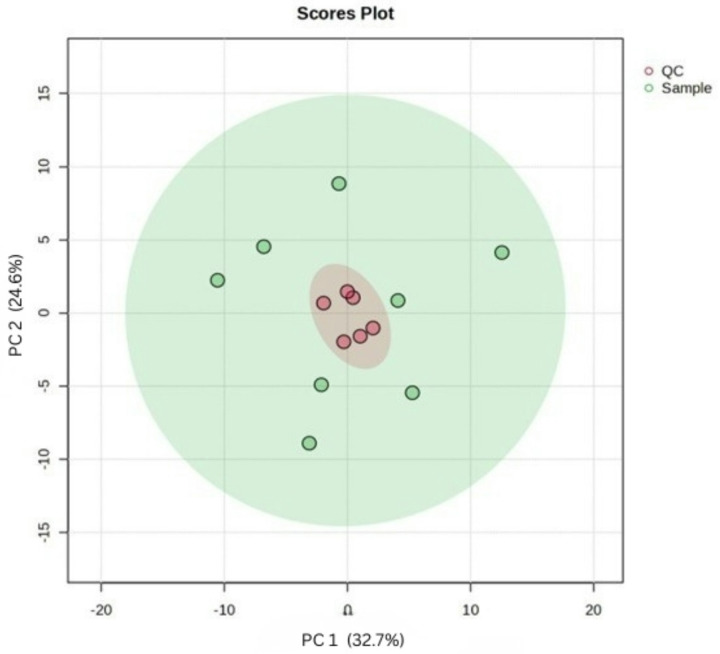
Principal component analysis score plot showing distinct clustering of quality control samples (QCs) and separation between BALF experimental groups (samples)

As indicated in Fig. 1, the consistent grouping of the QC samples within that of the experimental samples, supports the reliability of the data and suggests that machine drift or analytical variability is absent. These observations afflrm the quality and reproducibility of the dataset, validating its suitability for downstream multivariate and univariate statistical analyses.

#### Chemometric analysis results

**Figure 2:**
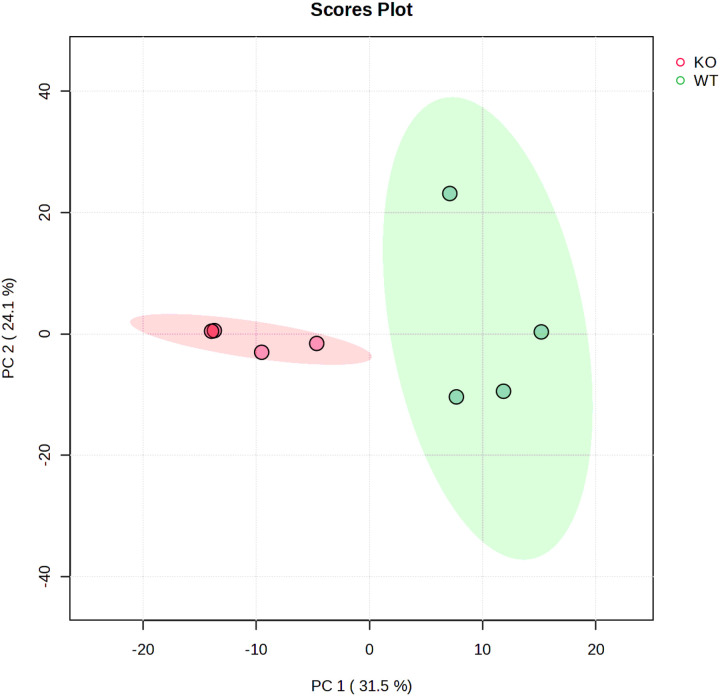
Principal component analysis score plot for the CysLT1 KO and WT sample groups. The percentages in brackets represent the proportion of variation explained in the observed data by the specific principal component (PC).

Figure 2. Principal component analysis score plot for the CysLT1 KO and WT sample groups. The percentages in brackets represent the proportion of variation explained in the observed data by the specific principal component (PC).

The clear separation between these groups along both PC1 and PC2 indicates significant metabolic differences between the CysLTR1 KO and WT groups.

#### Statistics and Metabolite Marker Selection

**Figure 3:**
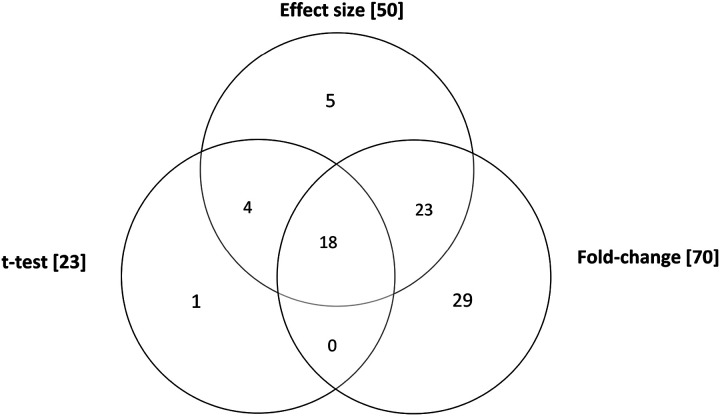
Venn diagram indicating the selection of the 18 metabolite markers contributing most significantly to the differences observed when comparing the CysLTR1-KO and WT BALF sample groups via a multistatistical approach. The selection criteria included metabolites having a t-test p-value < 0.05, a Log2FC of threshold 0.5, and an effect size ≥ 0.8.

Of the aforementioned 201 metabolites in the final data set, 23 metabolites showed a significant t-test p-value of < 0.05, 70 metabolites a log2FC threshold beyond 0.5, and 50 metabolites a Cohen’s d ≥ 0.8, when comparing the groups. The 18 metabolite markers that best describe the variance between CysLTR1 KO-deficient and WT BALF samples were selected via the aforementioned multistatistical selection approach as illustrated in the Venn diagram in Fig. 3.

Table 1 presents the metabolites that most significantly differ when comparing the CysLTR1 KO and WT BALF groups.

## Discussion

**Figure 4:**
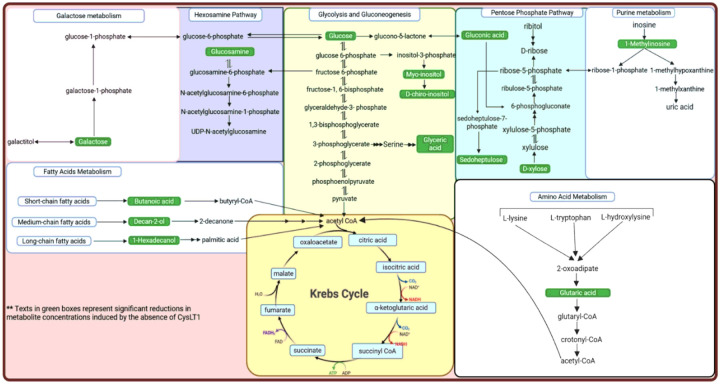
Schematic summary of the most significantly altered metabolites (highlighted in green) shown to be reduced in the lung BALF samples due to the absence of CysLTR1.

Figure 4 shows a schematic summary of those metabolites most significantly changed in the mice BALF samples collected from the lungs, due to the absence of CysLTR1 (highlighted in green), when compared to the WT mice. The altered pathways in the CysLTR1 KO included changes to glycolysis, the pentose phosphate pathway, purine metabolism, amino acid metabolism, fatty acid metabolism, galactose metabolism, and the hexosamine biosynthesis pathway.

The metabolomic analysis of biofluids such as serum, plasma, urine, cerebrospinal fluid, and BALF is a powerful tool for understanding the physiological and pathological states of disease conditions and, in this case, that of the lung (Evans et al., 2014; Stringer et al., 2016). In clinical research, providing a comprehensive snapshot of metabolic alterations in a biological system / sample /tissue, is important for identifying disease biomarkers, elucidating changes to biochemical pathways, and monitoring therapeutic responses (Dar et al., 2023; Perakakis et al., 2018). The results of the present investigation provide useful insights into the biochemical and physiological changes associated with CysLT1 and its receptor, further highlighting the potential role of CysLT1 in pulmonary physiology and disease mechanisms, as revealed by changes in the metabolic pathways influenced by the absence of CysLTR1 in the KO mice (Fig. 4).

The most important finding of this study is the significantly reduced in glucose (and galactose) in the BALF of CysLT1 KO mice comparatively, confirming a previously reported association between CysLTs and pulmonary glucose homeostasis (Filgueiras et al., 2015; Mothe-Satney et al., 2012). Furthermore, Guo et al., (2018) reported reduction in glucose-stimulated insulin secretion in MIN6 cells, a mouse pancreatic beta-cell line, via CysLTR1. In the absence of CysLTR1, insulin secretion is improved, resulting in improved systemic glucose clearance and glucose uptake into insulin-dependent tissues, including skeletal muscle, adipose tissue and the heart. This phenomenon results in reduced glucose availability to other organs that take up glucose via non-insulin-dependent pathways, including the lung, and hence results in reduced glucose levels in the BALF of CysLTR1 KO mice as seen in this study. This observation provides important insight into the role of CysLTR1 in regulating the metabolic microenvironment of the lung, particularly under inflammatory conditions. CysLTR1, a receptor for the leukotrienes LTC_4_, LTD_4_, LTE_4_ and LTB_4_ (Emala, 2018), plays a critical role in mediating inflammatory responses in the lung (Figueroa et al., 2001; Zhu et al., 2012). The absence of CysLTR1 results in a disruption of the normal leukotriene-mediated signaling cascade, and subsequently a reduced inflammatory cell response in the airways. Since the activation of various immune cells (M1 macrophages, neutrophils and T lymphocytes) results in a metabolic reprogramming and a heavy reliance on glycolysis, which subsequently consume substantial amounts of glucose during inflammation (Pająk et al., 2024; Soto-Heredero et al., 2020), the observed reduction in glucose levels in the BALF of CysLTR1 KO mice may also be due to a reduced basal pulmonary inflammatory response resulting from the disruption of leukotriene-mediated signaling.

Although glucose is a fundamental energy source and a key metabolite in glycolysis, it also serves as a substrate for the pentose phosphate pathway (PPP), hexosamine pathway, and tricarboxylic acid (TCA) cycle. Hence, the reduced levels of glucose in this study led to downstream metabolic perturbations, as observed in the significant reduction of several carbohydrate-related metabolite intermediates, including myo-inositol, D-chiro-inositol, glyceric acid, glucosamine, gluconic acid, and sedoheptulose, all of which are directly or indirectly linked to glucose metabolism (Fig. 4).

Myo-inositol and D-chiro-inositol are isomeric forms of inositol, a sugar alcohol involved in cellular growth and insulin functionality (DiNicolantonio & H O’Keefe, 2022). Both metabolites are synthesized from glucose-6-phosphate, as shown in Fig. 4. In the absence of an adequate glucose supply, the availability of glucose-6-phosphate is limited, leading to reduced inositol biosynthesis (Lepore et al., 2021). Since myo-inositol is the precursor of D-chiro-inositol, a further reduction in the latter supports this finding. The depletion of these intermediates not only indicates impaired glucose metabolism (Lepore et al., 2021) but also a reduced insulin signal transduction (DiNicolantonio & H O’Keefe, 2022) and an altered membrane phospholipid composition, particularly in tissues such as the lung and liver (Suliman et al., 2022). Subsequently, the roles of these metabolites in type 2 diabetes mellitus has been widely investigated. Myo-inositol has been reported to inhibit glucose absorption in the intestine and promote muscle glucose uptake in rats, whereas clinical trials have demonstrated that both myo-inositol and D-chiro-inositol possess insulin-mimetic properties and the ability to improve insulin sensitivity in metabolic conditions associated with insulin resistance in humans (Chukwuma et al., 2016; Jeon et al., 2016).

Glucosamine is a precursor for glycosaminoglycans and glycoprotein synthesis, macromolecules that play crucial roles in various biological processes, including cell signaling, tissue development, and disease progression (Boullanger et al., 1990; Linhardt & Toida, 2004; Smock & Meijers, 2018; Tian & Zhang, 2013). The reduction of glucosamine in the absence of CysLTR1 in the KO group in this study, would result in impaired protein glycosylation, which in turn would impact pulmonary function, as glycosylation is crucial for maintaining mucosal barrier integrity and immune responses.

The PPP functions by synthesizing nucleotide precursors, including ribose-5-phosphate (Fig. 4), and maintaining a balance between NADP^+^ and NADPH (Stincone et al., 2015; TeSlaa et al., 2023). Since inflammatory cells rely on the PPP to support rapid proliferation and anabolic metabolism, reduced inflammation and glucose supply to the PPP results in diminished PPP flux, which is consistent with the reduction in the levels of some PPP-associated metabolites, such as D-xylose, gluconic acid and sedoheptulose in the CysLTR1 KO mice in this study. D-xylose is metabolized through the nonoxidative branch of the PPP, where it is interconverted via intermediates such as xylulose-5-phosphate to eventually produce ribose-5-phosphate. Similarly, gluconic acid (derived from glucose) also serves as a precursor for ribose-5-phosphate (Stincone et al., 2015; TeSlaa et al., 2023). The reduced availability of glucose, D-xylose and their resulting PPP precursors limits the capacity of the PPP to generate ribose-5-phosphate, which is required for nucleotide synthesis. Sedoheptulose metabolism functions in NADPH generation, the latter of which is primarily used towards reductive biosynthesis reactions, and for ribose synthesis, for nucleotide biosynthesis (Perl et al., 2011). The resulting decrease in nucleotide synthesis is further substantiated by the reduced levels of 1-methylinosine (Log2FC = − 1.182, effect size = 1.643, and p = 0.088]), a methylated purine nucleoside involved in purine metabolism, in the CysLTR1 KO mice in our study.

1-Hexadecanol and butanoic acid (Fig. 4), both intermediates of fatty acid metabolism, were also reduced in BALF of the CysLTR1 KO mice comparatively. This indicates a metabolic adaptation to compensate for lower glucose availability, thereby increasing the utilization of fatty acid derivatives for energy in the lung. Furthermore, as previously mentioned leukotrienes are lipid mediators derived from arachidonic acid (Di Gennaro & Haeggström, 2012), and play a key role in inflammatory processes, and disruptions in arachidonic acid pathways have been previously associated with changes in lipid metabolism (Zhang et al., 2023).

Glutaric acid is a metabolic product of lysine and tryptophan metabolism (Sauer et al., 2005). These amino acids are catabolized to glutaryl-CoA, which is then converted into crotonyl-CoA and eventually enters the TCA cycle (Trefely et al., 2020), possibly indicative of an altered amino acid metabolism. However, it is more likely, considering also the reduction in mandelic acid (Ji et al., 2022), tricarballylic acid (TA) (Russell & Forsberg, 1986) and decan-2-ol (Riley et al., 2012), that the aforementioned glutaric acid is due an altered intestinal microbiome (Cai et al., 2023;) in the CysLTR1 KO mice. Recent studies have previously associated these metabolites to microbial activity and dysbiosis under inflammatory conditions (Cai et al., 2023; Yu et al., 2025). Although there is as yet no direct link between CysLTR1 and the gut or intestinal microbiota, G protein-coupled receptors, the most abundant class of cell surface receptor, and to which CysLTR1 belongs, are known to interact with gut microbiota, influencing various physiological processes (Aleti et al., 2023). Therefore, the observed reduction in these metabolites most likely results from changes to microbial metabolism associated with the loss of CysLTR1-mediated inflammatory signaling, which may indirectly modulate the host–microbiome interface, and various inflammatory mediators, including leukotrienes, have been reported to influence the composition and activity of microbial communities (Carlos H. C. Serezani et al., 2005).

## Conclusion

CysLTR1 plays a key role in a variety of metabolic pathways necessary for maintaining lung homeostasis, the most significant of which is glucose uptake and metabolism, with consequential changes to lipid, PPP, nucleotide and microbiome metabolism. These observed changes underscore the central role of CysLT1 in insulin signaling and the inflammatory response, as suggested by previous studies (Guo et al., 2018).

CysLTR1 expression is reportedly increased in the airway mucosa of asthma patients, particularly during exacerbations (Zhu et al., 2012), and an important regulator of mucus secretion, eosinophil recruitment, and airway inflammation, which are all hallmarks of asthma. This study serves as a basis towards a better understanding of the role of CysLTR1 in the lung, and many such studies to follow could be used towards inflammation related lung disease prevention or improved therapeutic strategies for such.

## Figures and Tables

**Table 1:** Metabolites with most significance when comparing the CysLTR1 KO and WT BALF sample groups.

Metabolite (PubChem ID)	Average concentration (ng/L) / (standard deviation) Wild type mice CysLTR1 KO mice		Log2FC (threshold 0.5)	Cohens (effect size: d-value ≥ 0.8)	t-test p-value < 0.05
Tricarballylic acid (14925)	2.650 (1.994)	0.792 (0.100)↓	−4.029	2.911	0.001
Glucosamine (739)	9,707 (10.121)	5,825 (8.381)↓	−2.45	1.958	0.026
Decan-2-ol (2737541)	1.117 (0.987)	0.120 (0.269)↓	−2.435	1.945	0.027
D-Chiro-Inositol (16216949)	125.524 (60.474)	56.153 (14.225)↓	−1.639	2.558	0.013
Glucose (5793)	2.118 (1.647)	0.780 (0.593)↓	−1.440	0.824	0.036
Mandelic acid (22943025)	52.300 (18.357)	38.746 (37.000)↓	−1.366	3.362	0.002
D-xylose (135191)	23.682 (19.270)	9.937 (0.002)↓	−1.713	1.307	0.033
Glutaric acid (90037731)	37.290 (17.780)	20.310 (5.905)↓	−1.343	2.186	0.008
Sedoheptulose (5459879)	19.677 (6.298)	12.138 (4.172)↓	−1.191	2.803	0.002
Glyceric acid (752)	55.715 (12.841)	31.324 (7.776)↓	−1.174	2.939	0.002
Gluconic acid (10690)	222.814 (134.281)	155.146 (80.397)↓	−1.435	1.624	0.022
1-Hexadecanol (2682)	38.672 (19.981)	22.849 (9.901)↓	−1.273	1.758	0.011
(R*,S*)-2,3 Dihydroxy butanoic acid (250402)	8.782 (3.632)	5.921 (1.384)↓	−1.101	2.605	0.006
Butanoic acid (16213394)	325.566 (117.085)	207.031 (36.553)↓	−0.981	2.737	0.007
Myo-inositol (440388)	1912.119 (865.363)	1398.837 (441.522)↓	−0.932	1.676	0.037
Galactose (441035)	25632.153 (6700.214)	20646.841 (2718.550)↓	−0.632	2.766	0.003

## Data Availability

Data are available on request from the corresponding author

## Abbreviations

BALF: Bronchoalveolar lavage fluid
BSTFA: O-bis (trimethylsilyl) trifluoroacetamide
CysLTR1: Cysteinyl leukotriene receptor 1
Cysltr1−/−: CysLTR1-deficient
Cysltr1−/+: Heterozygous
CysLTs: Cysteinyl leukotrienes
GC-TOFMS: Gas chromatography-time of flight mass spectrometry
IL-6: Interleukin 6
KO: Knockout
LTB4: Leukotriene B4
NF-κB: Nuclear factor kappa B
NIST: National Institute of Standards and Technology
PCA: Principal component analysis
PPP: Pentose phosphate pathway
QT: Quality control
TA: Tricarballylic acid
TCA: Tricarboxylic acid
TMCS: Trimethyl chlorosilane
TMS: Trimethylsilyl
TNF-α: Tumor necrosis factor-α
VIP: Variable of importance in projection
WT: Wild-type

## References

[1] Aleti G., Troyer E. A., & Hong S. (2023). G protein-coupled receptors: A target for microbial metabolites and a mechanistic link to microbiome-immune-brain interactions. Brain, Behavior, and Immunity – Health, 32, 100671–100686. Elsevier Inc. 10.1016/j.bbih.2023.100671 PMC10407893 37560037

[2] Boullanger P., Jouineau M., Bouammali B., Lafont D., & Descotes G. (1990). The use of IV-alkoxycarbonyl derivatives of 2-amino-2-deoxy-D-glucose as donors in glycosylation reactions. Carbohydrate Research, 202, 151–164. Elsevier Science Publishers B.V. 2224888 10.1016/0008-6215(90)84077-8

[3] Cai S., Lin J., Li Z., Liu S., Feng Z., Zhang Y., (2023). Alterations in intestinal microbiota and metabolites in individuals with Down syndrome and their correlation with inflammation and behavior disorders in mice. Frontiers in Microbiology, 14, 1016872–1016886. 10.3389/fmicb.2023.1016872 36910172 PMC9998045

[4] Carlos H. C., Serezani D. M., Aronoff S., Jancar P., Mancuso, & Marc Peters-Golden. (2005). Leukotrienes enhance the bactericidal activity of alveolar macrophages against Klebsiella pneumoniae through the activation of NADPH oxidase. Blood, 106, 1067–1077. 15718414 10.1182/blood-2004-08-3323 PMC1895163

[5] Chukwuma C. I., Ibrahim M. A., & Islam M. S. (2016). Myo-inositol inhibits intestinal glucose absorption and promotes muscle glucose uptake: a dual approach study. Journal of Physiology and Biochemistry, 72, 791–801. 10.1007/s13105-016-0517-1 27601253

[6] Coffey M. J., Torretti B., & Mancuso P. (2015). Adipokines and Cysteinyl Leukotrienes in the Pathogenesis of Asthma. Journal of Allergy, 2015, 1–9. 10.1155/2015/157919 PMC4684870 26770217

[7] Dar M. A., Arafah A., Bhat K. A., Khan A., Khan M. S., Ali A. (2023, March 1). Multiomics technologies: Role in disease biomarker discoveries and therapeutics. Briefings in Functional Genomics, 22, 76–96. Oxford University Press. 10.1093/bfgp/elac017 35809340

[8] Di Gennaro A., & Haeggström J. Z. (2012). The Leukotrienes: Immune-Modulating Lipid Mediators of Disease. In Advances in Immunology, 116, 51–92. Academic Press Inc. 10.1016/B978-0-12-394300-2.00002-8 23063073

[9] DiNicolantonio J. J., & H O’Keefe J. (2022). Myo-inositol for insulin resistance, metabolic syndrome, polycystic ovary syndrome and gestational diabetes. Open Heart, 9, e001989. 10.1136/openhrt-2022-001989 35236761 PMC8896029

[10] Emala C. W. (2018). Pulmonary pharmacology. Pharmacology and Physiology for Anesthesia. 613–628. Elsevier. 10.1016/B978-0-323-48110-6.00030-2

[11] Evans C. R., Karnovsky A., Kovach M. A., Standiford T. J., Burant C. F., & Stringer K. A. (2014). Untargeted LC-MS metabolomics of bronchoalveolar lavage fluid differentiates acute respiratory distress syndrome from health. Journal of Proteome Research, 13, 640–649. 10.1021/pr4007624 24289193 PMC4068805

[12] Figueroa D. J., Breyer R. M., Defoe S. K., Kargman S., Daugherty B. L., Waldburger K., (2001). Expression of the Cysteinyl Leukotriene 1 Receptor in normal human lung and peripheral blood leukocytes. American journal of respiratory and critical care medicine, 163, 226–233. www.atsjournals.org. 11208650 10.1164/ajrccm.163.1.2003101

[13] Filgueiras L. R., Serezani H., C., & Jancar S. (2015). Leukotriene B4 as a potential therapeutic target for the treatment of metabolic disorders. Frontiers in Immunology, 6, 515. 10.3389/fimmu.2015.00515. Frontiers Media S.A. 26500652 PMC4597104

[14] Gromski P., Xu Y., Kotze H., Correa E., Ellis D., Armitage E., (2014). Influence of Missing Values Substitutes on Multivariate Analysis of Metabolomics Data. Metabolites, 4, 433–452. 10.3390/metabo4020433 24957035 PMC4101515

[15] Guo R., Jiang J., Jing Z., Chen Y., Shi Z., & Deng B. (2018). Cysteinyl leukotriene receptor 1 regulates glucose-stimulated insulin secretion (GSIS). Cellular Signalling, 46, 129–134. 10.1016/j.cellsig.2018.02.002 29412178

[16] Jeon Y., Aja S., Ronnett G. V., & Kim E. K. (2016). D-chiro-inositol glycan reduces food intake by regulating hypothalamic neuropeptide expression via AKT-FoxO1 pathway. Biochemical and Biophysical Research Communications, 470, 818–823. 10.1016/j.bbrc.2016.01.115 26802467

[17] Ji J., Zhang S., Tang L., Zhang M., Yuan M., Wang P., & Gao X. (2022). Integrative analysis of fecal metabolome and gut microbiota in high-fat diet-induced hyperlipidemic rats treated with Rosa Roxburghii Tratt juice. Journal of Functional Foods, 90, 104978. 10.1016/j.jff.2022.104978

[18] Kalidhindi R. S. R., Ambhore N. S., & Sathish V. (2021). Cellular and Biochemical Analysis of Bronchoalveolar Lavage Fluid from Murine Lungs. In Methods in Molecular Biology, 2223, 201–215. Humana Press Inc. 10.1007/978-1-0716-1001-5_15 33226597 PMC7780416

[19] Kanaoka Y., & Boyce J. A. (2004). Cysteinyl Leukotrienes and Their Receptors: Cellular Distribution and Function in Immune and Inflammatory Responses. The Journal of Immunology, 173, 1503–1510. http://journals.aai.org/jimmunol/article-pdf/173/3/1503/1186911/1503.pdf 15265876 10.4049/jimmunol.173.3.1503

[20] Koller A., Bruckner D., Aigner L., Reitsamer H., & Trost A. (2020). Cysteinyl leukotriene receptor 1 modulates autophagic activity in retinal pigment epithelial cells. Scientific Reports, 10(1). 10.1038/s41598-020-74755-w PMC7573618 33077798

[21] Lepore E., Lauretta R., Bianchini M., Mormando M., Di Lorenzo C., & Unfer V. (2021). Inositols depletion and resistance: principal mechanisms and therapeutic strategies. International Journal of Molecular Sciences, 22, 6797. 10.3390/ijms22136796 34202683 PMC8268915

[22] Linhardt R. J., & Toida T. (2004). Role of glycosaminoglycans in cellular communication. Accounts of Chemical Research, 37, 431–438. 10.1021/ar030138x 15260505

[23] Liu X. R., Li M., Hao Q. Q., Yu Y. J., Liao C., Yu R., Kong D. L., & Wang Y. (2025). Unraveling cysteinyl leukotrienes and their receptors in inflammation through the brain-gut-lung axis. Virulence, 16,(1). 10.1080/21505594.2025.2502555 PMC12077450 40351036

[24] Mosala P., Mpotje T., Abdel Aziz N., Ndlovu H., Musaigwa F., Nono J. K., & Brombacher F. (2024). Cysteinyl leukotriene receptor-1 as a potential target for host-directed therapy during chronic schistosomiasis in murine model. Frontiers in Immunology, 15, 1279043. 10.3389/fimmu.2024.1279043 38840916 PMC11150569

[25] Mothe-Satney I., Filloux C., Amghar H., Pons C., Bourlier V., Galitzky J., (2012). Adipocytes secrete leukotrienes: Contribution to obesity-associated inflammation and insulin resistance in mice. Diabetes, 61(9), 2311–2319. 10.2337/db11-1455 22688342 PMC3425405

[26] Neves J. A. J., De Matos M. R., Ramalho T., Santos-Bezerra D. P., Cavalcante C. D. G. D., Peixoto A., R. D., (2020). Increased leukotriene B4 plasma concentration in type 2 diabetes individuals with cardiovascular autonomic neuropathy. Diabetology and Metabolic Syndrome, 12, 99. 10.1186/s13098-020-00606-3 33292560 PMC7663893

[27] Pająk B., Zieliński R., & Priebe W. (2024, March 1). The Impact of Glycolysis and Its Inhibitors on the Immune Response to Inflammation and Autoimmunity. Molecules, 29, 1298. 10.3390/molecules29061298 38542934 PMC10975218

[28] Pang Z., Lu Y., Zhou G., Hui F., Xu L., Viau C., (2024). MetaboAnalyst 6.0: towards a unified platform for metabolomics data processing, analysis and interpretation. Nucleic Acids Research, 52, 398–406. 10.1093/nar/gkae253 PMC11223798 38587201

[29] Perakakis N., Yazdani A., Karniadakis G. E., & Mantzoros C. (2018, October 1). Omics, big data and machine learning as tools to propel understanding of biological mechanisms and to discover novel diagnostics and therapeutics. Metabolism, 87, A1–A9. 10.1016/j.metabol.2018.08.002 30098323 PMC6325641

[30] Perl A., Hanczko R., Telarico T., Oaks Z., & Landas S. (2011, July). Oxidative stress, inflammation and carcinogenesis are controlled through the pentose phosphate pathway by transaldolase. Trends in Molecular Medicine, 17, 395–403. 10.1016/j.molmed.2011.01.014 21376665 PMC3116035

[31] Riley R. T., Torres O., Showker J. L., Zitomer N. C., Matute J., Voss K. A., (2012). The kinetics of urinary fumonisin B1 excretion in humans consuming maize-based diets. Molecular Nutrition and Food Research, 56, 1445–1455. 10.1002/mnfr.201200166 22815244 PMC3820424

[32] Russell J. B., & Forsberg N. (1986). Production of tricarballylic acid by rumen microorganisms and its potential toxicity in ruminant tissue metabolism. British Journal of Nutrition, 56, 153–162. 10.1079/bjn19860095 3676191

[33] Sabounchi-Schütt F., Åström J., Hellman U., Eklund A., & Grunewald J. (2003). Changes in bronchoalveolar lavage fluid proteins in sarcoidosis: A proteomics approach. European Respiratory Journal, 21, 414–420. 10.1183/09031936.03.00060902 12661994

[34] Salimi M., Stöger L., Liu W., Go S., Pavord I., Klenerman P., Ogg G., & Xue L. (2017). Cysteinyl leukotriene E4 activates human group 2 innate lymphoid cells and enhances the effect of prostaglandin D2 and epithelial cytokines. Journal of Allergy and Clinical Immunology, 140(4), 1090–1100. 10.1016/j.jaci.2016.12.958 28115217 PMC5624780

[35] Sánchez-Galán E., Gómez-Hernández A., Vidal C., Martín-Ventura J. L., Blanco-Colio L. M., Muñoz-García B., (2009). Leukotriene B4 enhances the activity of nuclear factor-κB pathway through BLT1 and BLT2 receptors in atherosclerosis. Cardiovascular Research, 81, 216–225. 10.1093/cvr/cvn277 18852255

[36] Sauer S. W., Okun J. G., Schwab M. A., Crnic L. R., Hoffmann G. F., Goodman S. I., (2005). Bioenergetics in glutaryl-coenzyme A dehydrogenase deficiency: A role for glutaryl-coenzyme A. Journal of Biological Chemistry, 280, 21830–21836. 10.1074/jbc.M502845200 15840571

[37] Smock R. G., & Meijers R. (2018). Roles of glycosaminoglycans as regulators of ligand/receptor complexes. Open Biology. 10.1098/rsob.180026. 8. PMC6223220 30282658

[38] Soto-Heredero G., Gómez de las Heras M. M., Gabandé-Rodríguez E., Oller J., & Mittelbrunn M. (2020). Glycolysis – a key player in the inflammatory response. FEBS Journal, 287, 3350–3369. Blackwell Publishing Ltd. 10.1111/febs.15327 32255251 PMC7496292

[39] Stincone A., Prigione A., Cramer T., Wamelink M. M. C., Campbell K., Cheung E., (2015). The return of metabolism: Biochemistry and physiology of the pentose phosphate pathway. Biological Reviews, 90, 927–963. 10.1111/brv.12140 25243985 PMC4470864

[40] Stringer K. A., McKay R. T., Karnovsky A., Quémerais B., & Lacy P. (2016). Metabolomics and its application to acute lung diseases. Frontiers in Immunology, 7, 44. 10.3389/fimmu.2016.00044 26973643 PMC4770032

[41] Suliman M., Case K. C., Schmidtke M. W., Lazcano P., Onu C. J., & Greenberg M. L. (2022). Inositol depletion regulates phospholipid metabolism and activates stress signaling in HEK293T cells. Biochimica et Biophysica Acta - Molecular and Cell Biology of Lipids, 1867. 10.1016/j.bbalip.2022.159137 PMC9070113 35247568

[42] Sullivan G. M., & Feinn R. (2012). Using Effect Size—or Why the P Value Is Not Enough. Journal of Graduate Medical Education, 4, 279–282. 10.4300/jgme-d-12-00156.1 23997866 PMC3444174

[43] TeSlaa T., Ralser M., Fan J., & Rabinowitz J. D. (2023). The pentose phosphate pathway in health and disease. Nature Metabolism, 5, 1275–1289. Nature Research. 10.1038/s42255-023-00863-2 PMC11251397 37612403

[44] Tian Y., & Zhang H. (2013). Characterization of disease-associated N-linked glycoproteins. Proteomics, 13, 504–411. 10.1002/pmic.201200333 23255236 PMC3733363

[45] Trefely S., Lovell C. D., Snyder N. W., & Wellen K. E. (2020, August 1). Compartmentalised acyl-CoA metabolism and roles in chromatin regulation. Molecular Metabolism,38. 10.1016/j.molmet.2020.01.005 PMC7300382 32199817

[46] Yu J., Liu C., Wang D., Wan P., Cheng L., & Yan X. (2025). Integrated microbiome and metabolome analysis reveals altered gut microbial communities and metabolite profiles in dairy cows with subclinical mastitis. BMC Microbiology, 25. 10.1186/s12866-025-03810-1 PMC11877966 40033186

[47] Zhang Y., Liu Y., Sun J., Zhang W., Guo Z., & Ma Q. (2023, October 1). Arachidonic acid metabolism in health and disease. MedComm, 4, e363. 10.1002/mco2.363 37746665 PMC10511835

[48] Zhu J., Bandi V., Qiu S., Figueroa D. J., Evans J. F., Barnes N., (2012). Cysteinyl leukotriene 1 receptor expression associated with bronchial inflammation in severe exacerbations of COPD. Chest, 142, 347–357. 10.1378/chest.11-1581 22871757 PMC3425162

